# Preterm, early term, and post-term infants from Riyadh mother and baby multicenter cohort study: The cohort profile

**DOI:** 10.3389/fpubh.2022.928037

**Published:** 2022-09-13

**Authors:** Amel Fayed, Hayfaa A. Wahabi, Samia Esmaeil, Hala Elmorshedy, Hilala AlAniezy

**Affiliations:** ^1^Clinical Sciences Department, College of Medicine, Princess Nourah Bint Abdulrahman University, Riyadh, Saudi Arabia; ^2^Research Chair of Evidence-Based Healthcare and Knowledge Translation, King Saud University, Riyadh, Saudi Arabia; ^3^Department of Family and Community Medicine, King Saud University Medical City and College of Medicine, Riyadh, Saudi Arabia

**Keywords:** preterm, early term, post-term, gestational age, Saudi Arabia

## Abstract

**Background:**

Birth before 37 or beyond 42 gestational weeks is associated with adverse neonatal and maternal outcomes. Studies investigating determinants and outcomes of these deliveries are scarce. The objective of this study was to determine the neonatal birth profile in relation to the gestational age at delivery and to evaluate its influence on the immediate maternal and neonatal outcomes.

**Methods:**

This is a multicenter cohort study of 13,403 women conducted in three hospitals in Riyadh. Collected data included sociodemographic characteristics, obstetric history, and physical and laboratory measurements. Regression models were developed to estimate the adjusted odds ratio (OR) and confidence intervals (CI) to determine factors associated with preterm, early term, and post-term births and to evaluate common maternal and neonatal risks imposed by deliveries outside the full term.

**Results:**

The incidence of preterm, early term, and post-term delivery was 8.4%, 29.8%, and 1.4%, respectively. Hypertensive events during pregnancy consistently increased the risk of all grades of preterm births, from more than 3-fold for late preterm (OR = 3.40, 95% CI = 2.21–5.23) to nearly 7-fold for extremely early preterm (OR = 7.11, 95% CI = 2.24–22.60). Early term was more likely to occur in older mothers (OR = 1.30, 95% CI = 1.13–1.49), grand multiparous (OR = 1.21, 95% CI = 1.06–1.38), pregestational diabetes (OR = 1.91, 95% CI = 1.49–2.44), and gestational diabetes women (OR = 1.18, 95% CI = 1.05–1.33). The risk of post-term birth was higher in primiparous. In preterm births, the adverse outcome of neonates having an APGAR score of <7 at 5 min and admission to neonatal intensive care units increased progressively as the gestational age decreased. Post-term births are 2-fold more likely to need induction of labor; meanwhile, preterm births were more likely to deliver by cesarean section.

**Conclusion:**

This large cohort study was the first in Saudi Arabia to assess the delivery profile across a continuum of gestational age and the associated maternal and neonatal adverse outcomes of deliveries outside the full-term period. The study showed that the prevalence of preterm and post-term birth in Saudi Arabia is similar to the prevalence in other high-income countries. The immediate adverse pregnancy outcomes inversely increased with the decrease in gestational age at delivery. In addition, maternal age, hypertension, diabetes, and parity influenced the gestational age at delivery.

## Introduction

Birth before 37 gestational weeks or beyond 42 gestational weeks has been the subject of investigation due to the many recognized poor immediate and late outcomes associated with such births (1, 2).

Recent reports estimated that 15 million babies are born preterm worldwide each year with the highest burden of the condition in low- and middle-income countries (LMIC) (3–5). Preterm birth (PTB) is the leading cause of death among children under 5 years of age with one million deaths annually; in addition, it accounts for 35% of mortality among those who are 1–28 days old (3, 6). Extreme PTB (≤28 weeks) is associated with marked respiratory and neuro-developmental disorders (7) with a decreasing gradient of risk of poor outcomes as the gestational age at delivery increases. Nevertheless, late PTB (32 to <37 weeks) is associated with repeated hospital admissions, longstanding illness, and poor general health at 3–5 years of age when compared to children born at term (2, 8). Most of the burden of PTB is due to babies born between 32 weeks and <37 weeks as they account for 86% of total PTB (3, 9), hence the high economic burden observed for healthcare provision for these children (10, 11).

A study from Saudi Arabia estimated the prevalence of PTB as 9% from the total birth (12), and another study reported on the outcomes of extreme PTB showed that more than 40% of these neonates died before discharge from the hospital, 36% of the survivors had cerebral palsy, and almost 40% were developmentally delayed (13). Both studies were from tertiary centers in Riyadh—the capital city, which may limit the generalization of the results to other parts of the Kingdom.

In 2015, there were 2.6 million third trimester stillbirths with 98% occurring in LMIC (14). Prolonged pregnancy was reported as a risk factor in 14% of total stillbirths, while other risk factors, such as congenital malformations, maternal infection, and malnutrition, accounted for only 6–7% each (14). The risk of stillbirth at 37 weeks is 11 per 100,000 as reported by a recently published systematic review of cohort studies (15). However, the same risk increases to 318 per 100,000 at 42 weeks of gestation (15). The prevalence of stillbirth in Saudi Arabia is 13/1,000 as recently estimated in a multicohort study from Riyadh, and only 1.5% of pregnancies have gone beyond 41 weeks gestation (12).

The objectives of this study are to determine the birth profile of Riyadh Mother and Baby Multicenter Cohort Study (RAHMA) neonates in relation to the gestational age at delivery and its influence on the immediate maternal and neonatal outcomes and the risk factors for preterm, early term, and post-term birth.

### Ethical consideration

This study was approved by the Institutional Review Boards at each participating hospital and followed Helsinki's Declaration guidelines for research on human subjects. Informed consent was obtained from all subjects involved in the study.

## Materials and methods

RAHMA study is a hospital-based prospective birth cohort study that was carried out in three hospitals in Riyadh, Saudi Arabia. Data were collected using a self-administered questionnaire inquiring about sociodemographic data and a standardized data extraction sheet to abstract all obstetric and laboratory data available in participating women's medical records. The study participants were recruited through a multistage stratified random sampling technique where the type of hospital was considered as the stratifying element (Ministry of Health hospitals, University hospitals, and military hospitals with the exclusion of private hospitals) and each hospital was considered as a whole cluster. Recruitment of the cohort started in November 2013, and data collection was completed in March 2015. Data collection tools were validated and developed after a thorough literature review, and both content and face validity were tested prior to usage. Validation was assured by a pilot study of the data collection tools (the questionnaire and the data extraction sheet), and extensive training of data collectors in the three participating hospitals was done to ensure that all data are collected in the same way. Content validation was confirmed by expert opinions, and panel discussions and literature reviews of relevant studies were done prior to the approval of the final version of data collection tools. Data extraction from medical records was performed by trained nurses in each participating hospital under the supervision of the charge nurse and research coordinators. A weekly random check of completeness and accuracy of data was done for quality assurance of collected data. Other details are available in the cohort profile of the study (12).

The aim of this study was to investigate the maternal and newborn outcomes of women recruited for the RAHMA multicenter cohort study according to gestational age at delivery. We considered 13,403 singleton pregnancies of women who delivered at ≥24 weeks of gestation from the RAHMA cohort in this report after exclusion of multiple pregnancies (*n* = 424) from the analysis, except for the total prevalence of PTB, missing gestational age (*n* = 383), or missing living status of the newborn (*n* = 358). We compared all gestational age groups to the reference group (39–41 gestational weeks) with respect to sociodemographic characteristics such as maternal age, educational attainment, working status, and exposure to secondhand smoke (SHS). In addition, maternal characteristics such as body mass index (BMI), associated chronic diseases such as hypertension and diabetes, and obstetric history elements, such as parity, were compared between the gestational age groups.

We investigated the influence of gestational age on the following maternal and neonatal outcomes: rate of induction of labor, mode of delivery normal vaginal birth, cesarean section (CS), neonatal admission to the intensive care unit (NICU), APGAR score at 5 min, and rate of stillbirth.

### Definitions

1. Gestational age at birth is defined as the time span between conception and birth of an infant, calculated from the last menstrual period and/or early ultrasound scan when there is a difference between menstrual date and ultrasound date; the latter was taken as the correct date. It is further classified as follows (16):
  - Term pregnancy 39–41 weeks
  - Post-term ≥ 42 weeks
  - Early term pregnancy 37–38 weeks
  - PTB is defined as infants born before completing the gestational age of 37 weeks that was further subdivided on the basis of gestational age into:
    ° Late preterm 34–36 weeks
    ° Early preterm 32–33 weeks
    ° Very early preterm 28–31 weeks
    ° Extremely preterm <28 weeks
2. Stillbirth: non-living birth at or after 28 weeks of gestation (14).
3. Maternal BMI was calculated from maternal weight at 28–30 weeks gestation and height with the following cutoff values as suggested by Catalano et al. (17): normal weight (≤28.4 kg/m^2^), overweight (28.5–32.9 kg/m^2^), and obese (≥ 33 kg/m^2^).
4. Gestational diabetes mellitus (GDM) should be diagnosed at any time in pregnancy according to World Health Organization guidelines (18) if one or more of the following criteria are met:
  - Fasting plasma glucose 5.1–6.9 mmol/L (92–125 mg/dL).
  - 1-h plasma glucose ≥10.0 mmol/L (180 mg/dL) following a 75 g oral glucose load.
  - 2-h plasma glucose 8.5–11.0 mmol/L (153–199 mg/dL) following a 75 g oral glucose load.
  - Pregestational diabetes (PGDM) is having type 1 or type 2 diabetes diagnosed before the index pregnancy.
5. Hypertensive events during pregnancy according to the report of the national high blood pressure (19): Pre-eclampsia is defined as new onset of elevated blood pressure after 20 weeks of pregnancy in a previously normotensive woman (≥140 mm Hg systolic or ≥90 mm Hg diastolic on at least 2 occasions 6 h apart) in addition to proteinuria of at least 1+on a urine dipstick or ≥300 mg in a 24-h urine collection. Eclampsia is defined as seizures in a pre-eclamptic woman that cannot be attributed to other causes. Gestational hypertension is defined as new onset of elevated blood pressure (≥140 mm Hg systolic or ≥90 mm Hg diastolic on at least two occasions 6 h apart) after 20 weeks of gestation in a previously normotensive woman and superimposed pre-eclampsia as new onset of pre-eclampsia after 20 weeks of pregnancy. Due to the low prevalence of these events, they were all aggregated as one variable for a more robust statistical analysis.

### Statistical analysis

We conducted a statistical analysis using IBM SPSS 26 software (SPSS, Chicago, IL, USA). All categorical variables were expressed as percentages and frequency, and all numeric variables were expressed as average ± standard deviation. The association between the incidence of prematurity and risk variables and outcomes was examined using chi-square analysis. Logistic regression models were adopted to investigate the effect of different maternal and neonatal factors on different stages of PTB. Adjusted odds ratios with their 95% confidence intervals (95% CI) were used to estimate the increased odds of preterm delivery associated with each risk factor. Modified logistic regression models (20) were used to estimate crude and adjusted risk ratios (RR), respectively, along with their 95% C.I. where women with full-term delivery were considered as the reference group, and confounders were determined according to their clinical significance in each outcome. The independent effect of gestational age on maternal and neonatal outcomes was evaluated by regression models considering women with full-term gestation as the reference group after adjustment of maternal age, parity, BMI, diabetes, and hypertension. A *p*-value below or equal to 0.05 is considered statistically significant.

## Results

We identified 13,403 singleton pregnancies of women who delivered at ≥22 weeks of gestation from the RAHMA cohort; the prevalence of PTB, including multiple deliveries, was 8.4% and 9.6% when multiple pregnancies were considered. Full-term deliveries accounted for 60.3% (*n* = 8,084), and early term deliveries constituted 29.8% of all deliveries, while post-term deliveries represented 1.4% of deliveries (*n* = 194). Late preterm incidence was 5.8% (*n* = 778), while early preterm was 1.1% (*n* = 148), very early preterm was 0.9% (*n* = 117), and extreme preterm was noted in only 0.6% (*n* = 86).

Maternal characteristics according to the gestational age at delivery are presented in Table 1. There was no statistically significant association between gestational age and maternal educational attainment, employment status, or exposure to secondhand smoke; however, maternal age, parity, BMI, diabetes, and hypertension were associated with the incidence of different gestational age categories (Table 1).

**Table 1:** Maternal demographic characteristics by gestational age at delivery.

	**≥42 Post term (*n* = 194)**	**39–41 Full-term (*n* = 8,084)**	**37–38 Early-term (*n* = 3,996)**	**34–36 Late preterm (*n* = 778)**	**32–33 Early preterm (*n* = 148)**	**28–31 Very early preterm (*n* = 117)**	**<28 Extreme preterm (*n* = 86)**	***P*-value**
**Age**								
<20 (*n =* 319)	5 (1.6)	192 (60.2)	92 (28.8)	16 (5.0)	5 (1.6)	4 (1.3)	5 (1.6)	<0.01
20–34(*n =* 9,938)	151 (1.5)	6,228 (62.7)	2,763 (27.8)	538 (5.4)	112 (1.1)	87 (0.9)	59 (0.6)	
35 or more (*n =* 3,146)	38 (1.2)	1,664 (52.9)	1,141 (36.3)	224 (7.1)	31 (1.0)	26 (0.8)	22 (0.7)	
**Education**								
Illiterate (*n =* 225)	3 (1.3)	123 (54.7)	80 (35.6)	14 (6.2)	1 (0.4)	3 (1.3)	1 (0.4)	0.82
Schools (*n =* 4,496)	70 (1.6)	2,718 (60.5)	1,351 (30.0)	254 (5.6)	41 (0.9)	36 (0.8)	26 (0.8)	
University/above (*n =* 3,216)	43 (1.3)	1,940 (60.3)	990 (30.8)	173 (5.4)	31 (1.0)	19 (0.6)	20 (0.6)	
**Working status**								
Housewife (*n =* 9,981)	155 (1.6)	6,054 (60.7)	2,948 (29.5)	576 (5.8)	109 (1.1)	77 (0.8)	62 (0.6)	0.38
Employee/student (*n =* 1,412)	16 (1.1)	830 (58.8)	456 (32.3)	80 (5.7)	11 (0.8)	11 (0.8)	8 (0.6)	
**BMI**								
Normal (*n =* 3,472)	43 (1.2)	2,065 (59.5)	1,007 (29.0)	238 (6.9)	51 (1.5)	38 (1.1)	30 (0.9)	<0.01
Overweight (*n =* 3,576)	51 (1.4)	2,221 (62.1)	1,015 (28.4)	209 (5.8)	30 (0.8)	29 (0.8)	21 (0.6)	
Obese (*n =* 4,455)	83 (1.9)	2,683 (60.2)	1,374 (30.8)	227 (5.1)	37 (0.8)	34 (0.8)	17 (0.4)	
**SHS**								
Yes (*n =* 8,360)	131 (1.6)	5,002 (59.8)	2,526 (30.2)	482 (5.8)	88 (1.1)	68 (0.8)	63 (0.8)	0.43
No (*n =* 2,474)	32 (1.3)	1,482 (59.9)	757 (30.6)	147 (5.9)	29 (1.2)	18 (0.7)	9 (0.4)	
**Parity**								
P1 (*n =* 2,971)	59 (2.0)	1,914 (64.4)	733 (24.7)	178 (6.0)	36 (1.2)	26 (0.9)	25 (0.8)	<0.01
P2-4 (*n =* 6,347)	84 (1.3)	3,937 (62.0)	1,849 (29.1)	336 (5.3)	56 (0.9)	50 (0.8)	35 (0.6)	
P5+ (*n =* 4,074)	51 (1.3)	2,227 (54.7)	1,410 (34.6)	263 (6.5)	56 (1.4)	41 (1.0)	26 (0.6)	
**Diabetes during pregnancy**								
Normal (*n =* 6,450)	98 (1.5)	4,026 (62.4)	1,854 (28.7)	342 (5.3)	60 (0.9)	46 (0.7)	24 (0.4)	<0.01
PRE-GDM (*n =* 394)	4 (1.0)	175 (44.4)	161 (40.9)	39 (9.9)	8 (2.0)	4 (1.0)	3 (0.8)	
GDM (*n =* 2,184)	29 (1.3)	1,260 (57.7)	732 (33.5)	110 (5.0)	16 (0.7)	21 (1.0)	16 (0.7)	
**Hypertensive events during pregnancy**								
No	192 (1.5)	7,887 (61.1)	3,814 (29.5)	719 (5.6)	131 (1.0)	97 (0.8)	77 (0.6)	<0.01
Yes	1 (0.3)	145 (37.5)	151 (39.0)	49 (12.7)	15 (3.9)	17 (4.4)	9 (2.3)	
Pre-existing hypertension	0(0.0)	59 (0.7	68 (1.7)	16 (2.1)	4 (2.7)	5 (4.3)	3 (3.5)	<0.01
Pregnancy-induced hypertension	1 (0.5)	79 (1.0)	71 (1.8)	23 (3.0)	8 (5.4)	7 (6.1)	4 (4.7)	<0.01
Pre-eclampsia	0 (0.0)	34 (0.4)	54 (1.4)	23 (3.0)	9 (6.2)	12 (10.4)	4 (4.7)	<0.01

BM, body mass index; SHS, secondhand smoking; PGDM, pregestational diabetes; GDM, gestational diabetes.

**Figure 1:**
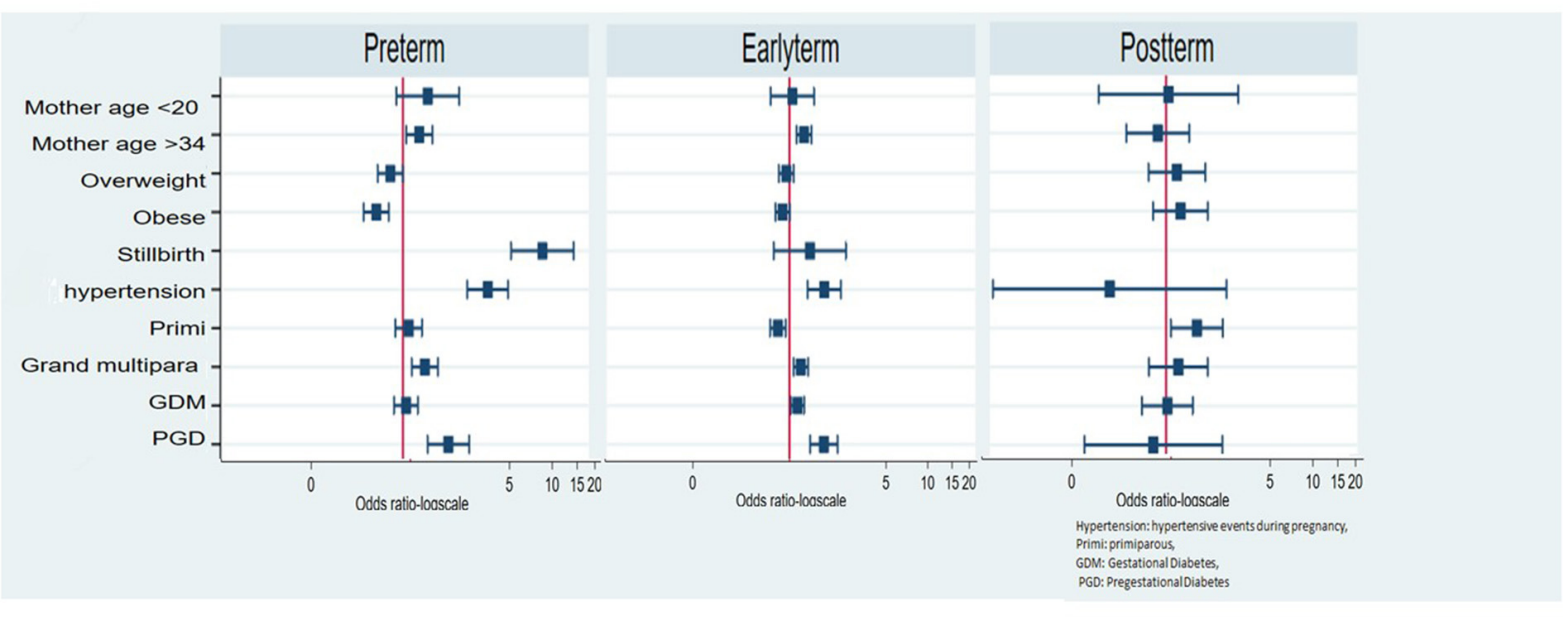
Independent effects of maternal and fetal factors on the gestational age at delivery.

Independent effects of maternal and fetal factors on the gestational age at delivery are illustrated in Table 2. The early term was more likely to occur in older mothers (35 years or more) (OR = 1.30, 95% CI = 1.13–1.49), grand multiparous (OR = 1.21, 95% CI = 1.06–1.38), women with pre-GDM (OR = 1.91, 95% CI = 1.49–2.44), women with GDM (OR = 1.18, 95% CI = 1.05–1.33), and women with any hypertensive events during pregnancy (OR = 1.81, 95% CI = 1.33–2.45), while it was less likely to be reported among primiparous women (OR = 0.81, 95% CI = 0.69–0.93) (Table 2 and Figure 1).

**Table 2:** Independent effect of maternal and fetal factors on gestational age at delivery.

	**37–38 Early-term (*n =* 3,996)**	**34–36 Late preterm (*n =* 778)**	**32–33 Early preterm (*n =* 148)**	**28–31 Very early preterm (*n =* 117)**	** <28 Extreme preterm (*n =* 86)**	**≥42 Post term (*n =* 194)**
**Maternal age**						
20–34 (*n =* 9,939)	Ref					
<20 (*n =* 276)	1.00 (0.67–1.49)	1.21 (0.59–2.47)	1.53 (0.45–5.17)	4.31 (1.20–15.45) *	1.14 (0.16–9.41)	1.01 (0.32–3.31)
35 or more (*n =* 3,146)	1.30 (1.13–1.49)*	1.49 (1.14–1.94)*	0.77 (0.39–1.54)	0.85 (0.38–1.87)	1.53 (0.68–3.46)	0.61 (0.33–1.12)
**BMI**						
Normal (*n =* 3,472)	Ref					
Overweight (*n =* 3,576)	0.96 (0.84–1.10)	0.86 (0.66–1.11)	0.55 (0.30–1.02)	0.61 (0.29–1.26)	1.12 (0.47–2.68)	1.23 (0.75–1.99)
Obese (*n =* 4,455)	0.89 (0.78–1.01)	0.64 (0.49–0.83)*	0.50 (0.28–0.91)*	0.533 (0.26–1.07)	0.59 (0.28–1.54)	1.25 (0.78–2.01)
**Parity**						
P1 (*n =* 2,971)	0.81 (0.69–0.93)*	1.06 (0.80–1.39)	1.32 (0.72–2.40)	0.88 (0.41–1.93)	1.60 (0.64–4.11)	1.71 (1.10–2.66)*
P2–4 (*n =* 6,347)	Ref					
P5+ (*n =* 4,074)	1.21 (1.06–1.38)*	1.37 (1.05–1.79)*	1.63 (0.86–3.09)	1.78 (0.85–3.75)	1.35 (0.50–3.63)	1.26 (0.76–2.09)
**Diabetes**						
Normal (*n =* 6,450)	Ref					
PGDM (*n =* 394)	1.91 (1.49–2.44)*	2.41 (1.62–3.61)*	1.41 (0.42–4.69)	1.53 (0.43–5.55)	1.97 (0.92–4.20)	0.83 (0.25–2.67)
GDM (*n =* 2,184)	1.18 (1.05–1.33)*	0.97 (0.77–1.24)	0.83 (0.44–1.54)	1.56 (0.82–2.92)	1.96 (0.29–5.76)	1.02 (0.66–1.57)
**Hypertensive events during pregnancy**						
No	Ref					
Yes	1.81 (1.33–2.45)*	3.40 (2.21–5.23)*	6.2 (2.69–14.36)*	8.71 (3.80–19.94)*	7.1 (2.24–22.60)*	0.4 (0.25–2.67)
**Stillbirth**						
No	Ref					
Yes	1.70 (0.86–3.36)	2.67 (0.96–7.41)	–	62.4 (27.06–144.30)*	119.5(49.7–287.3)*	-

BMI, body mass index; PGDM, pregestational diabetes; GDM, gestational diabetes.

The post-term delivery risk was significantly increased among primiparous women as compared to multiparous women (OR = 1.71, 95% CI = 1.10–2.66), and all other factors were not associated with post-term birth (Table 2 and Figure 1).

Hypertensive events during pregnancy consistently increased the risk of all grades of PTB, from more than 3-fold for late preterm (OR = 3.40, 95% CI = 2.21–5.23) to nearly 7-fold for extremely early preterm (OR = 7.11, 95% CI = 2.24–22.60). In addition, mothers with pre-GDM showed higher odds to have late preterm (OR = 2.41, 95% CI = 1.62–3.61) while GDM did not increase the risk of PTB. Grand multiparous women showed higher odds to have late preterm (OR = 1.37, 95% CI = 1.05–1.79) when compared to multiparous women (Table 2 and Figure 1).

Mothers who were obese were less likely to have late or early PTB when compared to mothers with normal BMI (Table 2 and Figure 1).

**Figure 2:**
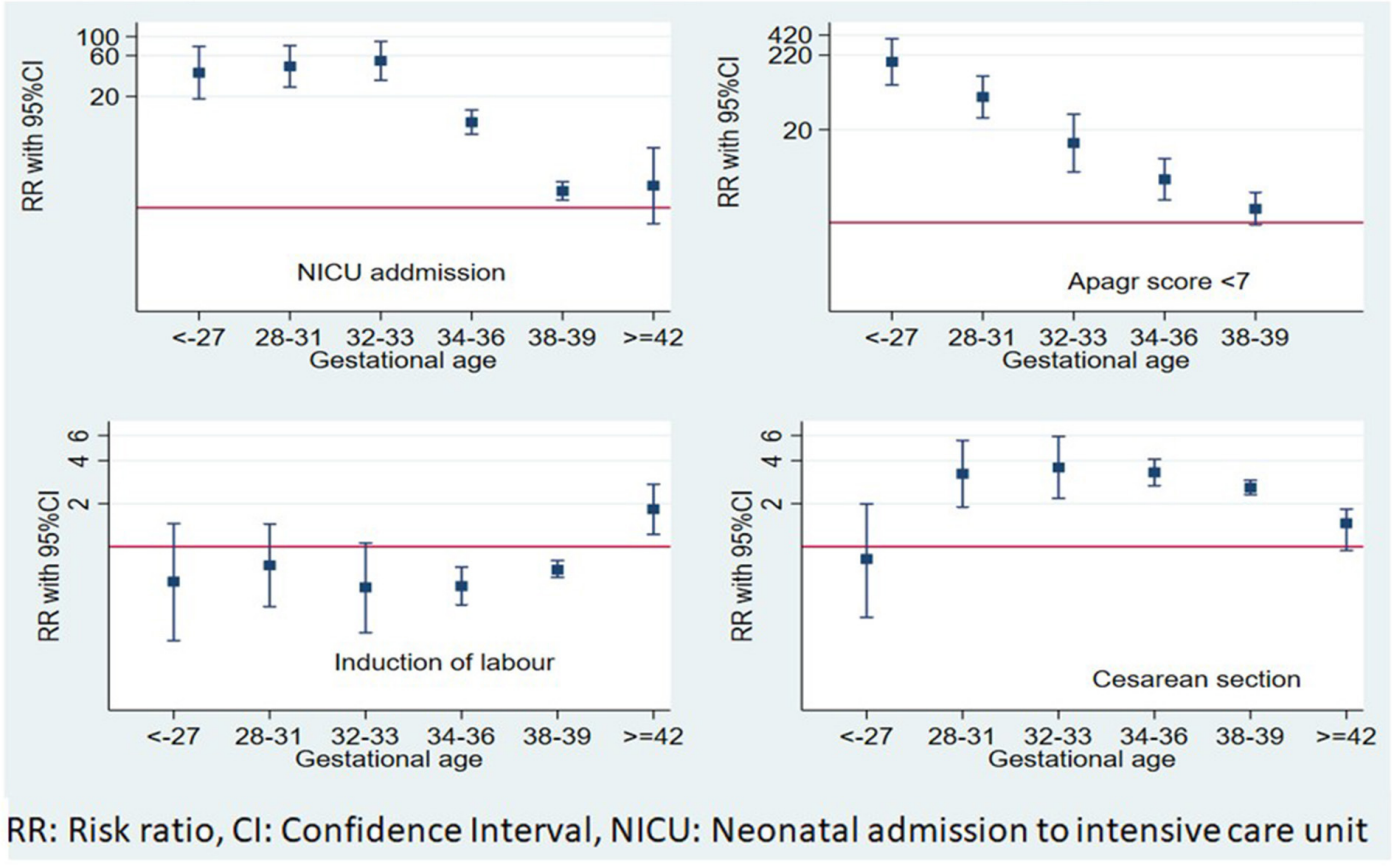
Independent effects of gestational age at delivery on maternal and short-term neonatal outcomes.

The independent effect of gestational age on maternal and neonatal outcomes is shown in Table 3 and Figure 2. Post-term births are 2-fold more likely to need induction of labor (RR = 1.83, 95% CI: 1.22–2.73); meanwhile, early term births as well as late, early, and very early PTB were more likely to deliver by CS.

**Table 3:** Independent effect of gestational age at delivery on maternal and short-term neonatal outcomes.

	**Crude risk ratio (95% CI)**	**Adjusted risk ratio (95% CI)**
**Induction of labor**
Early-term (37–38 WKS)	0.76 (0.68–0.85)*	0.69 (0.61–0.80)*
Late preterm (34–36 WKs)	0.66 (0.53–0.82)*	0.53 (0.39–0.72)*
Early preterm (32–33 WKs)	0.68 (0.41–1.11)	0.52 (0.25–1.06)
Very early preterm (28–31 WKs)	0.83 (0.49–1.39)	0.74 (0.38–1.44)
Extreme early preterm (<28 WKs)	0.94 (0.52–1.67)	0.56 (0.22–1.45)
Post term (42 Wks+)	1.87 (1.36–2.56)*	1.83 (1.22–2.73)*
**Cesarean section**
Early-term (37–38 WKS)	2.64 (2.42–2.88)*	2.59 (2.31–2.92)*
Late preterm (34–36 WKs)	2.91 (2.49–3.41)*	3.31 (2.67–4.09)*
Early preterm (32–33 WKs)	3.30 (2.36–4.61)*	3.59 (2.18–5.91)*
Very early preterm (28–31 WKs)	4.12 (2.85–5.96)*	3.24 (1.89–5.53)*
Extreme early preterm (<28 WKs)	1.41 (0.85–2.53)	0.82 (0.32–1.99)
Post term (42 Wks+)	1.17 (0.82–1.68)	1.46 (0.94–2.28)
APGAR <7
Early-term (37–38 WKS)	1.43 (0.96–2.14)	1.56 (0.93–2.62)
Late preterm (34–36 WKs)	5.76 (3.71–8.98)*	4.03 (2.07–7.83)*
Early preterm (32–33 WKs)	23.22 (13.67–39.43)*	12.93 (5.10–32.76)*
Very early preterm (28–31 WKs)	54.16 (33.38–87.88)*	57.06 (29.11–111.82)*
Extreme early preterm (<28 WKs)	133.76 (80.61–221.94)*	177.29 (84.35–372.95)*
Post term (42 Wks+)	0.73 (0.10–5.27)	-
**Admission to NICU**
Early-term (37–38 WKS)	1.62 (1.27–2.07)*	1.57 (1.23–2.01)*
Late preterm (34–36 WKs)	10.33 (8.05–13.25)*	10.02 (7.23–13.88)*
Early preterm (32–33 WKs)	42.16 (29.28–60.71)*	52.04 (30.88–87.67)*
Very early preterm (28–31 WKs)	51.89 (34.55–77.94)*	44.92 (25.72–78.47)*
Extreme early preterm (<28 WKs)	32.83 (20.36–52.93)*	37.92 (18.72–76.82)*
Post term (42 Wks+)	1.67 (0.73–3.81)	0.65–5.02)

WKS, weeks; NICU, neonatal intensive care unit. All groups were compared to full-term deliveries (39–41 weeks) as the reference group.

*P < 0.05. These models are adjusted for maternal age, maternal body mass index, parity, diabetes, and hypertension.

APGAR score <7 and admission to NICU risks increased progressively with the decrease in gestational age at delivery. Late PTB had the lowest risk, where the risk of APGAR <7 increased by 4-fold (RR = 4.03, 95% CI: 2.07–7.83) and admission to NICU risk nearly doubled (RR = 1.67, 95% CI: 1.23–2.26). Newborns of early term deliveries did not show an increased risk of APGAR <7 (RR = 1.56, 95% CI: 0.93–2.62), while they showed a significantly increased risk of admission to the NICU, (RR = 1.57, 95% CI: 1.23–2.01), compared to full-term infants.

## Discussion

This study showed that the prevalence of PTB, early term, and post-term delivery for singleton pregnancy was 8.4, 29.8, and 1.4%, respectively, while the prevalence of PTB among the whole cohort including multiple pregnancy was 9.6%. Hypertensive events during pregnancy increased the risk of all PTB; meanwhile, grand multiparity and PGDM increased the risk of late PTB. Older mothers have an increased risk of late PTB, while teenage mothers are at high risk of very early PTB. Stillbirths are more likely to be delivered prematurely. Post-term birth was more likely to occur among primiparous mothers. Mothers who were obese were less likely to have late or early PTB when compared to mothers with normal BMI.

The adverse outcomes of PTB including APGAR <7 and admission to the NICU risk increased progressively with the decrease in gestational age. In this study, induction of labor risk increased in post-term deliveries, while CS was significantly associated with PTB and early term birth as compared to full-term birth.

The prevalence of PTB reported in this study is comparable to the prevalence in other high-income countries such as Canada, Germany, and Qatar, which were 7.8%, 9.2%, and 8.8%, respectively (1, 3, 5). Prior to this report, only two studies from Saudi Arabia, published more than 10 years ago, reported on the prevalence of PTB with an estimation of between 7.5 and 8.2% which is comparable to the prevalence reported in this study (21, 22). Although a reduction in the prevalence of PTB is expected with the improvement of health services provision, in the period of time between previous publications and this study, the main risk factors for PTB in Saudi Arabia are not readily modifiable such as congenital malformations.

The etiology of PTB is an interplay of many medical and socioeconomic factors (23); hence, it differs between communities and ethnic groups (23, 24). Similar to previous reports, this study showed that pre-existing maternal diabetes, the occurrence of hypertensive events during pregnancy, and intrauterine fetal death were the main risk factors for PTB (1, 25). Some of the socioeconomic factors examined in this study were not associated with PTB; however, in a recently published study from Saudi Arabia, such factors were found to play an important role in the etiology of PTB including exposure to secondhand tobacco smoke, low family income, and first-degree relatives marriage (26). These factors are similar to those reported as a risk of PTB in other communities.

In agreement with previous studies from Saudi Arabia and other parts of the world, our results showed an inverse relationship between gestational age at delivery and neonatal survival and complications of prematurity (1, 27, 28). These complications included an increased rate of low APGAR scores and admission to the NICU; in addition, significantly, more preterm infants were delivered by CS. The increased need for intensive care for preterm infants is not surprising, considering the need for respiratory and metabolic support for the immature respiratory system, and liver and immune system of preterm infants (29, 30). CS delivery before term may be indicated for maternal reasons such as maternal hypertension or pre-eclampsia/eclampsia (31) and complications of maternal diabetes, or for fetal reasons such as fetal distress (32). In this study, the maternal indication for CS delivery of hypertension and diabetes was significantly more prevalent in the PTB category of infants.

There are conflicting reports about the association of increased perinatal morbidity and mortality with early term elective delivery (37 to <39 weeks), compared to late term elective delivery (39–40 weeks) (33, 34), which, in turn, is associated with increased risk of stillbirth at term (35).

The risk factors for early term delivery in this study included older maternal age, grand multiparity, and pregnancies complicated with PGDM, GDM, and hypertensive events during pregnancy, which were reported by other researchers (1). This profile of advanced maternal age with coexisting medical conditions explains the need for early elective delivery for maternal or fetal indications. Although maternal comorbidities contribute to neonatal morbidities associated with early term delivery, such as hypoglycemia, jaundice, and admission to NICU, nevertheless early term delivery is an independent risk for these adverse neonatal outcomes (36). These results are similar to our finding of increased risk of CS delivery and admission to NICU in this category of infants.

Contrary to the results of a recently published systematic review, which showed an association between maternal obesity and pot-term pregnancy (37), we did not find this association in our cohort.

Published reports showed that post-term birth is associated with an increased risk of maternal complications, including postpartum hemorrhage, shoulder dystocia, perineal tear, and CS delivery (38–40). In addition, babies who were born post-term have increased morbidities such as meconium aspiration, fetal distress, traumatic injury, and perinatal mortality (40, 41). However, there is no strong evidence for the benefits of induction of labor at term to avoid these complications (42). In this cohort, we did not find any significant association between the abovementioned complications and post-term birth, except for a higher rate of induction of labor. However, our findings may have been modified with the policy of offering induction of labor to all mothers who completed 41 gestational weeks, a common practice in the units where this study was conducted, hence the small number of subjects with post-term delivery in this study. A similar prevalence of post-term delivery was reported in other places in the world including China (43); nevertheless, due to the large number of subjects studied, the complications of post-term birth were significantly higher than those reported in our study.

We are aware of the limitations of this study as we did not investigate the full set of risk factors for PTB such as socioeconomic factors and consanguinity. In addition, we did not explore all the neonatal outcomes apart from those immediately found at delivery of the baby such as intrauterine growth restriction. However, this study is an important report on the RAHMA birth cohort documenting the main risk factors and immediate outcomes of a large population of neonates in Riyadh. Future studies of the RAHMA birth cohort will address in detail the influence of gestational age on pregnancy outcomes.

## Conclusion

This large cohort study was the first in Saudi Arabia to assess the delivery profile across a continuum of gestational ages and the associated maternal and neonatal adverse outcomes of deliveries outside the full-term period. The study showed that the prevalence of PTB and post-term birth in Saudi Arabia is similar to the prevalence in other high-income countries. The immediate adverse pregnancy outcomes inversely increased with the decrease in gestational age at delivery and maternal age and parity influence gestational age at delivery.

## Data availability statement

The raw data supporting the conclusions of this article will be made available by the authors, without undue reservation.

## Ethics statement

The studies involving human participants were reviewed and approved by the Institutional Review Boards at each participating hospital and followed Helsinki's declaration guidelines for research on human subjects. King Abdullah International Medical Research Centre, approval letter 11/062; King Fahad Medical City Research Centre, approval letter 013–017; and King Saud University, approval letter 13–985. The patients/participants provided their written informed consent to participate in this study.

## Author contributions

AF and HW were responsible for the study conception, design, and analysis plan. AF conducted the statistical analysis. HW, SE, HE, and HA drafted the manuscript. All authors read the final draft of the manuscript and approved it.

## Funding

This research was funded by the Deanship of Scientific Research at Princess Nourah Bint Abdulrahman University (grant number 41-PRFA-P-46). The funder did not play any role in the collection of data, the decision to publish, or the preparation of the manuscript.

## Conflict of interest

The authors declare that the research was conducted in the absence of any commercial or financial relationships that could be construed as a potential conflict of interest.

## Publisher's note

All claims expressed in this article are solely those of the authors and do not necessarily represent those of their affiliated organizations, or those of the publisher, the editors and the reviewers. Any product that may be evaluated in this article, or claim that may be made by its manufacturer, is not guaranteed or endorsed by the publisher.

## Abbreviations

LMIC: low- and middle-income countries
PTB: preterm birth
RAHMA: Riyadh Mother and Baby Multicenter Cohort Study
SHS: secondhand smoke exposure
BMI: body mass index
CS: cesarean section
NICU: neonatal admission to intensive care unit
GDM: gestational diabetes mellitus
PGDM: pregestational diabetes
OR: odds ratio
RR: risk ratio
CI: confidence interval.
